# Influence of Perfluorooctane Liquids in the Formation of Sticky Silicone Oil

**DOI:** 10.1155/2022/8434102

**Published:** 2022-02-04

**Authors:** Hirotsugu Takashina, Akira Watanabe, Tadashi Nakano

**Affiliations:** ^1^Department of Ophthalmology, Tokyo Rosai Hospital, 4-13-21 Omori-minami Ota-ku, Tokyo 143-0013, Japan; ^2^Department of Ophthalmology, The Jikei University School of Medicine, 3-25-8 Nishi-Shimbashi Minato-ku, Tokyo 105-8461, Japan

## Abstract

**Purpose:**

To examine the influence of perfluorooctane (PFO) in the formation of sticky silicone oil (SO).

**Methods:**

We performed in vitro experiments using PFO, SO, aqua, and canola oil (CO). The surface tension of CO relative to aqua is very close to that of SO or PFO. First, each material (0.5 ml) was carefully injected into the bottom of a transparent container that was filled with either aqua or CO. Next, a second material (0.5 ml) with a specific gravity that was lower than that of the first material was carefully injected onto the first material.

**Results:**

When the first material was injected into the container, the shape of the aqua was found to be close to a sphere, while the shapes of SO or PFO were prolate spheroids. Subsequently, when the second material was injected onto the first material, SO and CO completely adhered to the PFO, with the created immiscible droplets exhibiting a smooth surface. However, aqua did not create any immiscible droplets due to absence of adhesion to PFO or SO.

**Conclusions:**

Sticky SO is composed of PFO and SO, which easily form an immiscible droplet due to the low interfacial tension.

## 1. Introduction

Silicone oil (SO) tamponade is performed during vitrectomy for severe vitreoretinal diseases (e.g., rhegmatogenous retinal detachment with a giant retinal tear or proliferative vitreoretinopathy). Although subsequent SO removal is desirable in order to avoid future complications in relation to SO tamponade (e.g., oil emulsification or glaucoma), development of “sticky SO” as a complication makes SO removal difficult due to the presence of adhesion to the posterior retina. Dresp et al. reported that prolonged SO tamponade duration was one of the factors affecting the occurrence of sticky SO [1]. In contrast, no significant correlation between the occurrence of sticky SO and the SO tamponade duration was reported in other articles [2, 3]. In fact, Fukumoto et al. reported a remarkable case in which sticky SO occurred during a vitrectomy that used an SO injection [4]. Thus, the influence of SO tamponade duration on the occurrence of sticky SO has yet to be definitively determined. On the other hand, all these previous articles reported that intraoperative handling of perfluorocarbon liquids (PFCLs), such as perfluorooctane (PFO) and perfluorodecalin (PFD), is the potential cause of sticky SO [1–4]. This is especially the case with regard to the handling of PFO, as this has been reported to have a tendency to cause sticky SO to a greater degree than that seen for PFD [2, 3]. Since Dresp et al. mentioned the influence of surface tension on the formation of sticky SO [1], we performed in vitro experiments to elucidate the relationship between interfacial tension and sticky SO, after we experienced a case of sticky SO.

## 2. Materials and Methods

**Figure 1:**
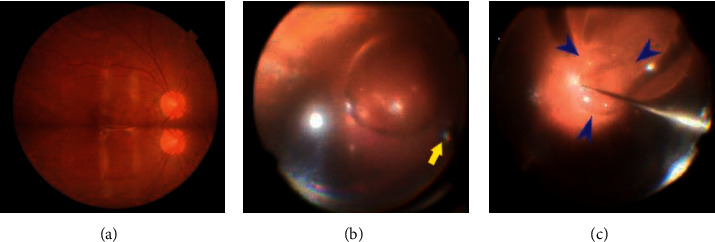
Fundus findings. A horizontal border crossing close to the macula (a) and sticky silicone oil on the posterior retina are shown. The needle of the Viscous Fluid Control Pak® (yellow arrow) was not able to reach the sticky silicone oil (b). Perfluorooctane was present between the silicone oil and posterior retina (area surrounded by blue arrowheads) (c).

A 53-year-old male underwent phacovitrectomy (using a 25-gauge system) for rhegmatogenous retinal detachment with a giant nasal retinal tear (from 12 to 5 o'clock) at Tokyo Rosai Hospital. During the surgery, PFO (PERFLUORON; Alcon Laboratories, Inc., Fort Worth, TX) was injected in order to flatten the detached retina before the performance of thorough vitreous shaving and endophotocoagulation around the retinal tear, and a direct exchange of PFO with SO (SILIKON^TM^ 1000; Alcon Laboratories, Inc., Fort Worth, TX) was performed at the end of the surgery. However, on postoperative day 1, we recognized a horizontal border crossing close to the macula during fundal examination performed with the patient in the seated position (Figure 1(a)). We speculated that the PFO remained in nearly half of the volume of the vitreous cavity, with intraoperative occurrence of severe corneal edema being responsible for this insufficient exchange. Due to the retinal toxicity of PFCL, we performed a second vitrectomy one week later in order to remove the PFO and SO using a Viscous Fluid Control Pak® (Alcon Laboratories, Inc., Fort Worth, TX) (VFC). In the middle of this PFO and SO removal, we found adhesion of the SO to the posterior retina (Figure 1(b)). The VFC needle was not able to reach the adhered SO due to the shortness of the needle, and we diagnosed the presence of sticky SO. At that time, the presence of a colorless and transparent fluid between the sticky SO and the posterior retina was recognized (Figure 1(c)), and it was inferred that the fluid was PFO due to the easy suction that was found when using the backflush needle. Subsequently, when the PFO was removed to approximately twice the optic disc diameter, the sticky SO was separated from the PFO and then ascended in the vitreous cavity. This made it possible to remove the SO using the VFC needle.

We speculated that adhesion of the SO to the retinal surface via PFO was the cause of the sticky SO in our case. So, we performed in vitro experiments using PFO, SO, aqua, and canola oil (CO).  Table 1 shows the surface tension, which is the interfacial tension between gas and liquid or solid, and the specific gravity of each material.  Table 2 shows the interfacial tension between the different liquids. The numerical values for the surface tension in Table 1 and the interfacial tension in Table 2 are rounded to the nearest integer.

The surface tension of CO relative to aqua is very close to that of SO or PFO, while SO and PFCL have similar characteristics, including being hydrophobic and lipophilic. Therefore, the characteristics of CO in relation to aqua are similar to those of PFO or SO. In the in vitro experiments, first, each material (0.5 ml) was carefully injected into the bottom of a transparent container that was filled with either aqua or CO. Next, a second material (0.5 ml) with a specific gravity that was lower than that of the first material was carefully injected into the first material.

## 3. Results and Discussion

**Figure 2:**
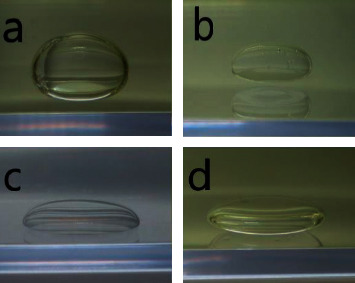
Shape of each material. Aqua in canola oil (a), silicone oil in canola oil (b), perfluorooctane in aqua (c), and perfluorooctane in canola oil (d). Aqua in canola oil was the closest in shape to a sphere (a), while all of the others were prolate spheroids.

The shapes of each first material are shown in Figure 2. The shape of aqua was found to be close to a sphere (Figure 2(a)), while the shapes of SO and PFO were prolate spheroids (Figures 2(b)–2(d)).

**Figure 3:**
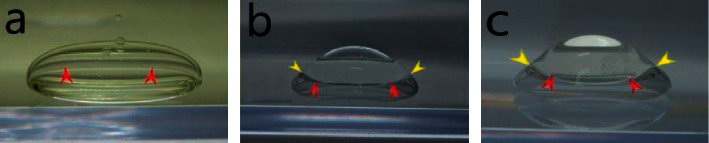
Shape of each immiscible droplet exhibiting a smooth surface. The shape of each immiscible droplet exhibiting a smooth surface is shown (the red arrowheads indicate the border between two materials). Silicone oil-perfluorooctane immiscible droplet in canola oil (upper: silicone oil, lower: perfluorooctane) (a), silicone oil-perfluorooctane immiscible droplet in aqua (upper: silicone oil, lower: perfluorooctane) (the yellow arrowheads indicate the contact point among perfluorooctane, silicone oil, and aqua) (b), and canola oil-perfluorooctane immiscible droplet in aqua (upper: canola oil, lower: perfluorooctane) (the yellow arrowheads indicate the contact point among perfluorooctane, canola oil and aqua) (c).

Subsequently, when the second material was injected into the first material, SO and CO completely adhered to the PFO, with the created immiscible droplets exhibiting a smooth surface. The shapes of each immiscible droplet are shown in Figure 3.

**Figure 4:**
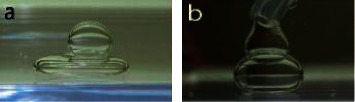
Lack of formation of an immiscible droplet with aqua in canola oil. Upper material: aqua; lower material: perfluorooctane (a). Upper material: silicone oil; lower material: aqua (b). The upper material immediately slid down the lower material after the picture was obtained (a, b).

However, the aqua did not create any immiscible droplets due to no adhesion to the PFO or SO (Figure 4), and the second material (Figure 4(a): aqua, Figure 4(b): SO) immediately slid down the first material (Figure 4(a): PFO, Figure 4(b): aqua) after the picture was obtained.

Since Dresp et al. mentioned the influence of surface tension in the formation of sticky SO [1], we performed these experiments using four materials (PFO, SO, aqua, and CO) to assess the adhesiveness between different liquids. CO contains more than 90% oleic acid + linoleic acid, having C-H, C = O, and C-OH bonds, SO contains Si-O bonds, and PFO contains C-F and C-H bonds. These chemical structures determine their surface tensions and interfacial tensions with water and with each other and also the shapes of the immiscible droplets. Since a higher surface tension indicates a stronger intermolecular force, there is matchlessly strongest intermolecular force for aqua among the four materials. Since the difference in intermolecular force between two materials influences the interfacial tension, the interfacial tension between SO and CO is matchlessly lower than the interfacial tensions between aqua and the other materials (Table 2). Similarly, the interfacial tensions between PFO and SO or CO are thought to be matchlessly lower than the interfacial tensions between aqua and PFO, SO, and CO.

We supposed that the matchlessly strongest intermolecular force in aqua was one of the reasons why the shape of aqua in CO was the closest to a sphere in the experiment (Figure 2(a)). In contrast, we supposed that the relatively large influence of the gravitation due to weak intermolecular force was one of the reasons why the shape of SO in CO was a prolate spheroid in the experiment (Figure 2(b)). In fact, the shapes of PFO in aqua (Figure 2(c)) and in CO (Figure 2(d)) were almost the same (spheroids), suggesting that the interfacial tension hardly influenced the shape of a single material. On the other hand, when a second material was injected into the first material, each immiscible droplet was composed of materials having a low surface tension (PFO, SO, and CO) (Figure 3), while aqua did not form any immiscible droplets with either PFO or SO in CO (Figure 4). Therefore, low intermolecular forces were inferred to influence the formation of immiscible droplets.

As described above, the interfacial tension had little influence of the shape of a single material. However, the shapes of the immiscible droplet composed of PFO and SO were considerably different, as seen in Figures 3(a) and 3(b), and the phenomenon might have been influenced by the material which was filled in the transparent container (Figure 3(a): CO; Figure 3(b): aqua). In fact, the contact point among the three immiscible liquids in Figure 3(a) (PFO, SO, and CO) was unclear, while the contact point among the three immiscible liquids in Figure 3(b) (PFO, SO, and aqua) was clear (yellow arrowheads in Figure 3(b)). In Figure 3(b), the interface between PFO and SO was roughly perpendicular to the interface between aqua and the immiscible droplet composed of PFO and SO at the contact point. The roughly perpendicular line at the contact point supports the suggestion that the interfacial tension between PFO and SO is much less than that between aqua and PFO or SO, based on the “Neumann's triangle” theorem, which states that the three vectors of interfacial tension at a contact point among three immiscible liquids are in balance [13]. As a result, PFO and SO easily form an immiscible droplet due to their low interfacial tension.

In our case, after the vitrectomy for PFO and SO removal, no further recurrence of rhegmatogenous retinal detachment was recognized. The PFO-SO immiscible droplet in the vitreous cavity was able to completely and closely attach over the entire giant retinal tear due to the superficial smoothness. This close attachment might be the reason why recurrence of rhegmatogenous retinal detachment was not recognized after PFO and SO removal. If this theory is correct, then the effectiveness of double SO tamponade [14] might be due to the superficial smoothness of the standard SO-heavy SO immiscible droplet.

There are some limitations to our current study. The first is that we used aqua instead of a balanced salt solution (BSS) during our in vitro experiments, since the correct surface tension of BSS has yet to be identified. Since the surface tension for a salt solution is higher than that for distilled water [15], the use of a BSS would have led to a large difference in the surface tension relative to PFO or SO. Thus, the use of aqua did not influence the current results. Second, we did not examine the adhesion between PFO and the retinal surface. In general, the retinal surface is uneven due to the presence of retinal vessels, foveal depression, and optic disc. In addition, it is possible that individual differences in the retinal vessels might exist. Therefore, a correct examination of the adhesion between PFO and the retinal surface is difficult. In anyway, because PFCL can be easily removed, adhesion between PFCL and SO rather than that between PFCL and the retinal surface is important to resolve sticky SO.

## 4. Conclusions

In conclusion, sticky SO is composed of PFO and SO, which easily form an immiscible droplet due to their low interfacial tension.

## Figures and Tables

**Table 1:** Specific gravity and surface tension of each material.

	PFO	SO	Aqua	CO
Specific gravity (g/ml)	1.75 [5]	0.97 [6]	1.0	0.91 [7]
Surface tension (dyne/cm)	17 [5]	21^*∗*^	72 [1, 8]	31 [9]

PFO = perfluorooctane, SO = silicone oil, CO = canola oil. *∗*Provided by Alcon Japan Ltd.

**Table 2:** Interfacial tension between different liquids.

	Aqua-PFO	Aqua-SO	Aqua-CO	SO-CO
Interfacial tension (dyne/cm)	40–45 [10]	35 [10]	33 [11]	2–3 [12]

PFO = perfluorooctane, SO = silicone oil, CO = canola oil.

## Data Availability

The datasets generated and/or analyzed during the current study are not publicly available due to limitations of ethical approval involving the patient data and anonymity, but are available from the corresponding author on reasonable request.
